# Electrodiagnostic Studies in the Surgical Treatment of Carpal Tunnel Syndrome—A Systematic Review

**DOI:** 10.3390/jcm10122691

**Published:** 2021-06-18

**Authors:** Katarzyna Osiak, Agata Mazurek, Przemysław Pękala, Mateusz Koziej, Jerzy A. Walocha, Artur Pasternak

**Affiliations:** 1 Medical Centre for Postgraduate Education, Department of Plastic Surgery, Professor W. Orlowski Memorial Hospital, 231st Czerniakowska Street, 00-416 Warsaw, Poland; kateosiak@gmail.com; 2 Department of Anatomy, Jagiellonian University Medical College, 12th Kopernika Street, 31-034 Krakow, Poland; agt.mazurek@student.uj.edu.pl (A.M.); pekala.pa@gmail.com (P.P.); mateusz.koziej@gmail.com (M.K.); jwalocha@poczta.onet.pl (J.A.W.)

**Keywords:** carpal tunnel release, carpal tunnel syndrome, median neuropathy, electrodiagnostic study

## Abstract

The aim of our paper was to provide comprehensive data on the role of electrodiagnostic (EDX) studies in the surgical treatment of carpal tunnel syndrome (CTS). An extensive search was conducted through the major electronic database to identify eligible articles. Data extracted included grade of CTS based on neurophysiological testing, preoperative data of EDX studies, time of complete or partial resolution after surgery, postoperative Boston carpal tunnel questionnaire (CTQ) scores, age, sex, intraoperative and postoperative data of EDX studies, time to complete or partial resolution of symptoms, and number of patients without postsurgical improvement. Our main findings revealed that that electrodiagnostic testing is still a powerful tool for diagnosis of CTS. Moreover, it can also detect other pathologies. EDX testing provides a quantitative measure of the physiological function of the median nerve, which may be used to guide surgical treatment. Thirdly, when the outcome of surgery is unsatisfactory, NCS can assist in determining the reason for failure.

## 1. Introduction

Carpal tunnel syndrome (CTS), first described in 1863 by Sir James Paget, is the most common focal entrapment mononeuropathy [1]. It is defined as a compression of the median nerve at the wrist associated with decreased function of the nerve at this level [2,3]. There are several risk factors of CTS including genetic heredity, diabetes mellitus, thyroid disease, obesity, rheumatoid arthritis, and pregnancy [4,5,6]. However, most cases of carpal tunnel syndrome are idiopathic [7]. CTS often affects persons of working age and may lead to absences from work and a marked decline in performance. Regardless of the risk factors, the prevalence of the disease in the general population is about 15% [8]. CTS constitutes the most expensive upper extremity musculoskeletal disorder in the United States, with costs exceeding $2 billion annually [9].

Primary diagnosis of CTS is based on characteristic clinical symptoms such as paresthesia (mainly numbness and tingling), pain at the wrist and hand and in severe cases weakness or atrophy of hand muscles. There are also some provocative tests for CTS, among which Phalen’s wrist flexion test, Tinel’s median nerve percussion test and Durkan’s carpal compression test are most commonly used in the clinical setting. In terms of investigations, electrodiagnostic (EDX) studies constitute a useful objective measure in CTS diagnosis [10]. EDX studies include nerve conduction studies (NCSs) and electromyography (EMG). NCSs confirm CTS by detecting impaired median nerve conduction across the carpal tunnel, with normal conduction elsewhere. EMG assesses pathologic changes in the muscles innervated by the median nerve, typically the abductor pollicis brevis muscle [11]. In cases with longstanding and severe compression, there will be evidence of denervation on EMG sampling of the abductor pollicis brevis muscle. 

Treatment options for CTS include wrist splinting, therapeutic ultrasound, local corticosteroid injections, and carpal tunnel release surgery [12]. Analysis of the costs of CTS treatment, including indirect non-healthcare costs such as loss of productivity caused by absence from work, led to the conclusion that established CTS is best treated with surgery [12]. Surgery has also been shown to be superior to non-operative treatment for patients with symptoms of CTS without changes due to denervation [13]. 

### EDX Testing Technique

The results of electrodiagnostic studies have been found to be highly sensitive and specific for the diagnosis of CTS. EDX testing is performed by using generally accepted standardized techniques according to the American Association of Neuromuscular and Electrodiagnostic Medicine (AANEM) summary statement [11]. In patients suspected of CTS, the following EDX studies are recommended: median sensory or mixed nerve conduction study, median motor conduction study, needle examination of APB, ulnar or/and radial motor and sensory NCSs (in order to exclude a peripheral neuropathy), and needle electromyography of the limb muscles innervated by the C5toT1 roots (in order to exclude a cervical radiculopathy, brachial plexopathy, and a proximal median neuropathy) be performed as part of the examination of patients suspected of CTS [14]. In cases of severe CTS in which the median sensory potentials and the compound muscle action potential (CMAP) from abductor pollicis brevis (APB) muscle are absent, a lumbrical-interosseous distal motor latency (DML) comparison is performed [15]. The severity of CTS is usually classified according to results obtained from the NCS which reflects the degree of demyelination and axonal loss in the median nerve [16]. 

Prolonged motor and sensory latencies of the median nerve and reduced sensory and motor conduction velocities in properly performed NCSs are accepted as diagnostic criteria for CTS. Many factors may influence the amplitude and latency of an individual nerve, giving a false-positive or false-negative result. Such factors include age, sex, finger diameter, concurrent systemic disease, obesity, and temperature. Applying a NCS as a relative comparison of two nerve segments, i.e., the median nerve and another nerve segment that does not travel through the carpal tunnel, could be useful. This technique is the most sensitive and accurate, with a sensitivity of 80–92% and specificity of 80–99% [10]. The electrophysiological classification of the severity of CTS has been defined by the American Association of Electrodiagnostic Medicine (AAEM) [17].

## 2. Aims

The aim of this systematic review is to

(1) provide comprehensive data on the role of electrodiagnostic (EDX) studies in the surgical treatment of carpal tunnel syndrome.
(2) review pre- and postoperative data on patients with CTS evaluated via EDX testing.
(3) analyze in detail patients baseline and preoperative clinical data with respect to electrophysiological condition.

Recent findings have revealed an absence of linear association between neurophysiological severity of the CTS and postoperative outcome, and thus there is a need to thoroughly explore pre- and postoperative clinical data to evaluate if EDX is still a powerful tool for diagnosing and developing treatment plans for carpal tunnel release [18,19,20,21,22].

## 3. Methods

### 3.1. Literature Search

A comprehensive database search was performed between May and August 2020 in PubMed using a combination of search terms: “carpal tunnel syndrome” AND “electrodiagnostic studies” OR “carpal tunnel syndrome” AND “nerve conduction studies”. Relevant publications were included for initial assessment. Duplications were eliminated and additional eligible articles from other sources (references of reviewed full-texts and from relative journals) were included. This study was performed in compliance with the Preferred Reporting Items for Systematic Reviews and Meta-Analyses (PRISMA) (Supplement) [23]. 

### 3.2. Eligibility Criteria

After initial an assessment of articles by title/abstract, all found full texts of included studies met the following inclusion criteria: (1) provided preoperative data (EDX results with comparison of clinical examination and/or CTQs (carpal tunnel questionnaires) scores of patients with carpal tunnel syndrome who underwent surgery) and (2) provided their postsurgical outcomes (with or without postoperative EDX results). Exclusion criteria were (1) patients diagnosed without EDX testing, (2) non-surgical treatment of CTS, (3) publications presenting only preoperative data, or (4) publications focused only on the diagnostic value of EDX testing. Letters to the editors, case reports, and conference abstracts were also excluded from the systematic review. Each publication was independently assessed by three authors (A.P., P.P., A.M.). Disagreements during the screening process were discussed and led to a consensus among the authors. 

### 3.3. Data Extraction

Data from final eligible articles were extracted and recorded by one of the searchers (A.M.) and collected data was independently checked and verified by two other authors (A.P., P.P.). Collected information included: grade of the CTS based on neurophysiological testing, preoperative data of EDX studies, time of complete or partial resolution after surgery, postoperative CTQs scores, age, sex, postoperative and intraoperative data of NCSs, time to complete or partial resolution of symptoms, and number of patients without postsurgical improvement.

### 3.4. Quality Assessment

The quality of 32 included studies was evaluated using the Newcastle–Ottawa scale (NOS) [24]. NOS comprises 9 items (‘stars’; each star for the most important factor) grouped into 3 sections: Selection (0–4 stars), Comparability (0–2 stars), Outcome (0–3 stars) for the assessment of cohort studies. Score 9 indicates the best quality of the publication, scores ≤5 indicate low quality. NOS scores of each included study are presented in Table 1.

## 4. Results

**Figure 1:**
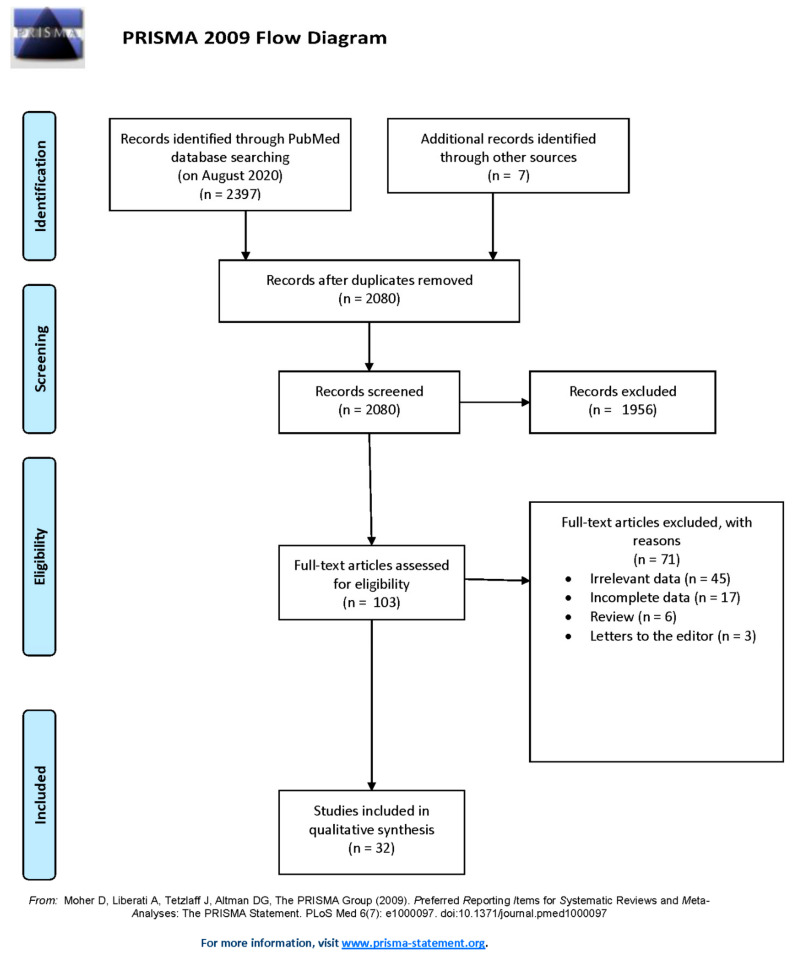
Flow-chart of systematic review.

A total of 2397 publications were identified by initial literature search. After additional records (*n* = 7) and removing duplications (*n* = 324), 2080 studies were included for initial assessment by title and/or abstract. Among those, 1956 publications were excluded after title/abstract screening, while 103 full texts were assessed for eligibility. Forty-five studies presenting irrelevant data and seventeen publications with reported incomplete data were excluded. Reviews and letters to the editor were also not included in the systematic review. A total of 32 of the initial 2080 publications identified were included in this study. A flow chart presenting the processing of articles through the study following the PRISMA protocol is presented in Figure 1 [23]. 

**Figure 2:**
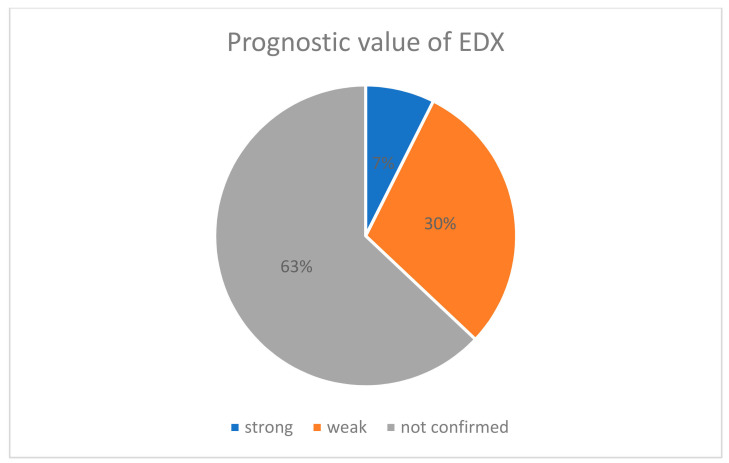
Percentage pie chart presenting prognostic value of EDX.

A total of 24 studies presented detailed pre- and postoperative data and extracted data of these publications were presented in Table S1 (supplement available online). The summary of Table 2 and graphic presentation of the prognostic value of EDX according to confirmed significant correlation between preoperative EDX and recovery after surgery (Figure 2) are included below:

According to our results, the majority of publications did not confirm the prognostic value of EDX (only 2 of 28 articles). Padua et al. [25,26] revealed a strong correlation between neurophysiological severity of CTS and recovery, while 8 of 28 publications suggest a weak prognostic value of the EDX.

### Bias Assessment

NOS scores of included publications are presented in Table 1. Mean NOS score (±SD) was 6.56 ± 1.34.

## 5. Discussion

Carpal tunnel syndrome is often but not invariably caused by a demonstrable median neuropathy at the wrist (MNW). Thus, there are patients with CTS with normal EDX and asymptomatic patients with MNW as detected by EDX. Hence, NCSs provide a reliable measure to document MNW, not CTS, i.e., to demonstrate the underlying lesion responsible for the symptoms and signs of CTS. They support the diagnosis of CTS, but cannot make it by themselves. Thus, the defined abnormalities of nerve conduction need not be symptomatic. Patients with abnormal NCSs but no symptoms probably should not be labeled as having CTS but rather as MMW cases [27]. A study by De Kleermaeker et al. demonstrates that most patients with clinically defined CTS and normal EDX results will benefit from carpal tunnel release [28].

While nerve conduction abnormalities commonly improve following successful decompression surgery, they often do not normalize [28,29]. It can therefore be difficult to attribute a nerve conduction abnormality to recurrence of nerve compression in a patient who had previously undergone decompression surgery, particularly if preoperative studies were not performed. This is one of a number of reasons why NCSs should be undertaken in all patients with suspected carpal tunnel syndrome prior to surgery. It is worth mentioning that during the past few years, ultrasonographic (US) confirmation of CTS diagnosis has proven to be an easily applicable, patient-friendly, low-cost test. However, US cannot replace EDX for confirmation of clinical diagnosis of CTS. An abnormal US test result has a high positive predictive value for abnormal EDX results in clinically defined CTS [30].

Electrodiagnostic studies have an important role in predicting the outcome of surgical decompression, disclosing other pathologies, particularly in atypical cases, and providing a useful baseline if patients do not improve after surgery. Clinical recovery was associated with preoperative EDX according to Padua et al., 1996, 1997; Padua’s electrophysiological scale was associated with postoperative functional status according to Malladi et al., 2009. Despite these findings, most publications reveal only weak or no association with clinical recovery. The slowing of SNCV may be the only manifestation of early CTS [26]. DML is a routine neurophysiological parameter in the diagnosis of CTS and can be easily obtained, but it has a low diagnostic sensitivity and a low prognostic value in no-operated hands with CTS [31]. However, it appears to be a useful preoperative indicator of the neurophysiological results that can be expected after surgical decompression of the median nerve. Data of Padua et al. suggest that when DML is still within normal limits at the time of surgery, there is an excellent possibility that full nerve function will be restored, and neurophysiological normalization usually parallels the disappearance of CTS symptoms. When the DML is 4 to 6 ms, the possibility of neurophysiological normalization appears to be around 50% at 6 months. Prolongation of DML over 6 ms is not associated with neurophysiological normalization. In patients with DML ranging from 4 to 6 ms and more, a dramatic reduction of CTS sensory symptoms is observed, with disappearance of nocturnal symptoms; nonetheless, in some cases sensory and/or motor deficits can still be present after surgery [28]. Some authors revealed predictive value of DML [32], but still there is lack of strong association according to other authors. 

Finestone et al. and Rivlin et al. observed that outcomes after carpal tunnel release (CTR) were not affected by preoperative EDX grade. As all EDX grades benefit from surgical treatment, EDX evaluation may not be necessary to define treatment in patients with uncomplicated carpal tunnel syndrome [33,34].

The American Association of Neuromuscular and Electrodiagnostic Medicine (AANEM) practice parameter for EDX in CTS and the Normative Data Task Force (NDTF) outlines EDX studies and reference values considered standard of care in patients with clinical suspicion of CTS. These studies are valid and reproducible in confirming median neuropathy at the wrist with a high degree of sensitivity (>85%) and specificity (95%). EDX confirms the diagnosis of median neuropathy at the wrist, evaluates its severity, determines its pathophysiology, (axon loss versus demyelination) and excludes cervical radiculopathy and coincidental ulnar nerve disease or polyneuropathy. Patients undergoing CTR without resolution of symptoms were identified as having an alternative diagnosis upon EDX (polyneuropathy, radiculopathy, motor neuron disease, spondylotic myelopathy, syringomyelia, and multiple sclerosis). This is confirmed also by Basiri et al., who conclude that EDX studies are often necessary for exclusion of other possible causes of symptomatology, such as cervical radiculopathy, brachial plexopathy, and a proximal median neuropathy [35]. 

During the past few years, ultrasonographic (US) confirmation of CTS diagnosis has been the subject of many studies for several reasons. It is executed by comparing cross-sectional area measurements of the median nerve obtained at the level of the carpal tunnel with those obtained at the level of the pronator quadratus muscle. According to Claes et al. US cannot replace EDX for confirmation of clinical diagnosis of CTS [30]. However, an abnormal US test result has a high positive predictive value for abnormal EDX result in clinically defined CTS. US might reveal relevant anatomic information preoperatively that rarely has a direct influence on treatment management of patients with CTS. US testing, taking morphometric data into account, does not have the same diagnostic value as EDX does in confirming CTS.

In the majority of cases, a careful history and physical examination are sufficient to make a clinical diagnosis of CTS and make initial treatment decisions [27,36,37]. However, many patients present with multiple problems and/or their presentation is atypical. An EDX examination can confirm the clinical impression of CTS, which is reassuring for both the patient and physician. Other patients may have coexisting or confounding diagnoses that EDX testing and consultation can help sort out. Additionally, it is important to add that EDX studies with high sensitivity and lower specificity (e.g., ring-finger method) are suspected to lead to more false-positive diagnoses, and there is a requirement to establish cut-off values for these methods [27,35,38]. Performing, interpreting, and reporting high-quality and evidence-based EDX testing can potentially lead to improved outcomes in many patients. Some authors presented prognostic value of individual EDX methods, e.g., second lumbrical-interossei nerve test (2L-IN) [39] which could also be a better alternative EDX method in analysis nerve conduction in patients with severe thenar atrophy (which is suspected to alter NCS results) [35,40,41].

While both traditionalists and contemporary thinkers agree that a diagnosis can be made based on clinical symptoms, traditionalists argue that EDX, also known as nerve conduction studies, is the gold standard for confirming a CTS diagnosis [42,43]. They further claim that other provocative tests like Tinel’s and Phalen’s are not reliable and that ultrasound cannot be used to make a diagnosis of CTS [44]. Another study revealed lower NPV of ultrasound studies in comparison with NCSs in patients with very low suspicion of the CTS [45]. The diagnostic validity of commonly used clinical tests is debated. Studies have shown the Phalen test to have a sensitivity ranging from 10% to 88% and specificity ranging from 47% to 100% [45], but some authors have revealed that there is a correlation between positive Phalen’s and Tinel’s signs and preoperative EMG results, but in the same study there was no confirmed significant correlation between these provocative tests and nerve conduction velocity [45,46]. Additionally, CTS can have an atypical presentation with symptoms that differ from those previously mentioned. Due to these factors, EDX testing is commonly utilized to confirm a diagnosis. More recently, the American Academy of Orthopedic Surgeons’ Clinical Practice Guidelines have indicated that EDX may be helpful but not required to establish the diagnosis of CTS [47].

There are data suggesting that EDX testing can provide some information regarding prognosis after surgery. Preoperative electrodiagnostic testing is useful in predicting symptom recovery after carpal tunnel surgery and the severity on preoperative nerve conduction studies predicts the rate of postoperative symptom resolution [48]. Electrodiagnostic studies should be obtained before surgery to confirm the diagnosis and estimate prognosis because patients with more severe CTS are less likely to experience complete recovery after surgery [26,32,34,45,46,49,50,51,52]. 

The observations of Bland et al. imply that NCS findings are shown to be of prognostic value for surgical carpal tunnel decompression [53]. Patients with absent sensory and motor responses, most of whom probably have degeneration of many median nerve fibers rather than conduction block, had the worst outcomes [18,25,26,34,39,50,52,53]. Patients with moderate neurophysiologic abnormalities had the best outcomes. Some authors have analyzed which factors have a significant impact on the outcomes of patients with CTS who are treated surgically. According to the results of a multivariate analysis, there is no association between preoperative NCS results (median to ulnar latency > 0.5 ms) and improvement after surgery. It also revealed that only sex and age are statistically significant preoperative factors [19]. Some findings reveal that younger patients more often report recurrence of symptoms, but their NCS results showed better nerve condition in comparison with older patients [20]. There are other data suggesting an absence of association between preoperative NCS results and outcome [21,36,53,54]. Additionally, there are numerous publications that confirm that there is a correlation between preoperative NCS results and postsurgical outcome, wherein patients with mild and moderate CTS achieved improvement and complete relief earlier than patients with severe CTS [18,25,26,32,34,39,46,52,53,55,56,57]. Immediate improvement (intraoperative increase of conduction velocity) after decompression is related to reversible metabolic damage in nerve fibers, and this phenomenon is observed in some cases of mild CTS [58]. Those with normal NCS had outcomes almost as poor as those at the opposite end of the severity scale, possibly because some of the neurophysiologically negative cases were mistaken clinical diagnoses.

Perhaps more surprising, then, is the fact that patients with mild CTS—especially those in whom sophisticated, newer neurophysiologic techniques are required to show any abnormality—had poorer outcomes than did those with nerve conduction results of middle severity [32,34,53,59,60]. 

Change of job was more often observed in patients without improvement of symptoms [59] and better results were noticed in groups of patients with light activity in comparison with patients performing types of work with higher number of repetitions and which require higher loads [22,59,61]. 

EDX study has a considerable associated cost [62], the procedure itself is uncomfortable for patients, and there is literature suggesting that it is not routinely required for the diagnosis of CTS [63]. The prevalence of surgeons who perform CTR without preoperative neurophysiological test varies depending on surgical specialty [63,64]. Furthermore, some authors suggest that clinical severity of the CTS based on score of carpal tunnel questionnaire score might be sufficient to predict NCS results [64] or postsurgical outcomes [65], and there are also data arguing against clinical prediction of NCS results [66]. Gomes et al. distinguished independent factors that predicted neurophysiological diagnosis of CTS, including BMI ≥ 30, nocturnal worsening of symptoms, and awakening or positive provocative tests, but the authors noted that basing diagnosis on the clinical picture alone is still insufficient to diagnose CTS correctly [67,68]. 

Some surgeons specializing in plastic surgery, orthopedics, or neurosurgery, who perform the procedure of carpal tunnel release, see no value in NCS as part of their routine management of CTS patients, while neurophysiologists believe that their tests do add clinical value and should be carried out in every patient with suspected CTS before surgery [25,32,69].

## 6. Conclusions

EDX study is not only a diagnostic test for CTS but can also detect other pathologies. Secondly, EDX study provides a quantitative measure of the physiological function of the median nerve, which may be used to guide surgical treatment and for prognosis. Thirdly, when the outcome of surgery is unsatisfactory, EDX testing can assist in determining the reason for failure.

## Figures and Tables

**Table 1:** NOS score of included studies.

Author	Selection	Comparability	Outcome
Schlagenhauff & Glasauer 1971	3	2	3
Grudberg et al., 1983	3	1	2
Luchetti et al., 1991	3	1	2
Seror 1992	4	2	3
Braun et al., 1994	2	1	1
Lang et al., 1995	2	1	3
Padua 1996	3	2	3
Glowacki et al., 1996	3	2	3
Concannon et al., 1997	3	1	2
Higgs et al., 1997	3	1	3
Padua et al., 1997	3	2	3
Choi et al., 1998	3	2	3
Nakamura et al., 1999	2	1	2
Dudley Porras et al., 2000	2	1	1
Mondelli et al., 2001	2	1	2
Finsen et al., 2001	2	1	2
Bland et al., 2001	2	1	1
Uchiyama et al., 2002	3	1	3
Kouyoumdjian et al., 2002	3	1	3
Borisch et al., 2003	3	2	3
Rotman et al., 2004	3	1	2
Schrijver et al., 2005	3	2	2
Tay et al., 2006	2	1	3
Ginanneschi et al., 2008	2	1	3
Malladi et al., 2009	3	1	3
Inukai et al., 2012	3	1	3
Tahririan et al., 2012	3	1	3
Beck et al., 2013	3	2	3
Fowler et al., 2015	3	1	3
Kronlage et al., 2015	3	2	3
De Kleermaeker et al., 2017	2	1	2
Rivlin et al., 2018	3	2	2
Mean ± SD	2.72 ± 0.52	1.34 ± 0.48	2.50 ± 0.67

**Table 2:** Pre- and postoperative clinical and electrophysiological evaluation of patients with CTS.

Author	Number of EDX Tests	Number of Tests with Significant Prognostic Value	Grading (No = 0, Yes = 1)	Prognostic Value (Strong = 2, Weak = 1, Not Confirmed = 0)
Schlagenhauff & Glasauer 1971	1	0	0	0
Grudberg 1983	1	0	0	0
Seror 1992	2	0	0	0
Braun 1994	2	0	1	0
Lange 1995	2	0	0	0
Padua 1996	3	2	1	2
Głowacki 1996	2	0	0	0
Concannon 1997	1	0	1	0
Padua 1997	3	3	1	2
Choi 1998	2	0	1	0
Nakamura 1999	4	0	1	0
Dudley Porras 2000	4	2	1	1
Mondelli 2001	5	1	1	1
Finsen 2001	1	0	1	0
Bland 2001	4	0	1	1
Kouyoumdjian 2002	3	0	1	0
Borisch 2003	2	0	0	0
Schrijver 2005	3	0	0	0
Tay 2006	2	0	1	0
Malladi 2009	2	1	1	1
Inukai 2012	5	1	1	1
Tahririan 2012	3	0	1	0
Beck 2013	1	0	1	0
Fowler 2015	1	1	1	1
Kronlage 2015	2	1	1	1
DeKleemaeker 2017	4	0	0	0
Rivlin 2018	1	1	1	1
Rivlin 2018	1	1	1	1
strong—significant confirmed prognostic value in all groups of patients				
weak—confirmed significant prognostic value in some groups				

## Supplementary Materials

The following are available online at https://www.mdpi.com/article/10.3390/jcm10122691/s1. Table S1 Pre- and postoperative clinical and electrophysiological evaluation of patients with CTS.
