# Supplementary material for: Electrodiagnostic Studies in the Surgical Treatment of Carpal Tunnel Syndrome—A Systematic Review
Source: J Clin Med. 2021 Jun 18;10(12):2691. doi: 10.3390/jcm10122691 (PMC8235020; doi:10.3390/jcm10122691)
Supplement: Supplementary file 1 [file jcm-10-02691-s001.zip › jcm-1234154-supplementary.pdf]

Supplementary Table S1. Pre- and postoperative clinical and electrophysiological evaluation of patients with CTS.

Author	Year	Grade and number of patients	EDX	Preoperative data	Relief of symptoms	No improvement	Statistical analysis	Summary
Schlagenhauff & Glasauer	1971	no grading, 20 patients (29 hands)	DML (25 hands had performed examination)	**Sensory symptoms** (20/20): dysesthesia 18/20, numbness 15/20, pain 18/20; **Motor symptoms** (17/20): muscle weakness 16/17, atrophy 3/17	15/25 treated hands had complete resolution during the follow-up period (3-36 months); 5/25 hands - persistent slight motor signs, 5/25 - persistent sensory signs	**Sensory symptoms** (3-24 months after surgery): dysesthesia 4/5, numbness 3/5, pain 5/5; **Motor symptoms**: muscle weakness 5/5, atrophy 1/5	no data	No statistical analysis was done Any significant change cannot be confirmed
Grudberg et al.	1983	no grading, 26 patients with normal preoperative electromyogram completed 12-month follow-up (32 hands)	electromyogram	numbness and tingling 32/32 ; night pain 28/32; decreased sensibility 18/32;	24/32 (75%) – complete relief 2/32 - intermittent numbness and tingling 4/32 - mild pain in the wrist on strenuous effort	2/32 hands; 1 patient – abnormal EMG postoperatively	no data	No statistical analysis was done Any significant change cannot be confirmed
Braun et al.	1994	no grading, 125 patients underwent EDX testing	DSL, NCV	no data	positive group: 60/75, negative group: 40/50	15/75 positive group; 10/50 negative group	**pooled two-tailed student t-test**	no significant improvement after 2 months no significant differences between measured values at each measured time intervals (α=0.05) none of groups reached total recovery until 4th month further significant recovery between 4th and 6th months
Lang et al.	1995	22 patients and 16 age-matched control subjects, no grading	DML, SNCV	**The sum score of the neurological deficit:** 11.3 ±08 (mean value±SD) **vibration thresholds at the index finger:** 4.2±1.2 μm; **thresholds of warmth** (5.59 ± 0.62 °C) **and cold** (7.11 ± 0.9°C) sensation on the index finger;	**The sum score of the neurological deficit:** 1 month: 2.1 ± 0.5; 3 months: 1.2 ± 0.4; 6 months:1.3 ± 0.5; 12 months: 0.6 ± 0.3; 18 months: 0.8 ±0.4) improved (no correlation between the assessed nerve functions - pre- and postoperative data); Significant improvement in values of	no data	**Comparison of data between the timings: Newman-Keuls test** (interval scale) **the Wilcoxon-Wilcox test** (categorical scale)**; Data of patients and control subjects:** t-test, **For correlations:** Pearson test correlation (r)/ Spearman rank correlation (Rs)	Median DML, SNCV, vibration threshold, warmth and cold perception significantly improved after operation There was no correlation between postoperative sum score of neurological deficit and assessed nerve functions, neither within the

					temperature and vibration thresholds.			corresponding intervals nor between pre- and postoperative data
Padua et al.	1996	37 hands, MILD- 6/37, MOD – 16/37, SEV – 15/37;	**1M** - median NCV thumb to wrist; **3M** - median NCV middle finger to wrist; DML - distal motor latency wrist to thenar eminence;	**MILD group** 6/6 hands- no motor or sensory deficit; **MOD group** 2/16 impairment of hand gripping; 3/16 hands sensory deficits, 9/16 hands - difficulties in fine hand movements; **SEV group** - 7/15 thenar eminence hypotrophy, 4/15 cases Phalen's test positive	**6th month**: 0/37 - nocturnal awakening, pain; 0/6 of MILD group, 2/16 of MOD group, 4/15 of SEV - light difficulties in fine hand movements; 5/15 of SEV group - motor deficits still observed, 2/15 of SEV group - sensory deficits still observed; **electrophysiological normalisation:** 6/6 MILD, 4/16 - MOD, 0/15 -SEV	**6th month** - 1/37, the same patient as above of MILD group;	**Assessment between differences of consecutive evaluations:** paired Student's test, Kolmogorov-Smirnoff (non parametric test).	MILD group: significant improvement in 1M and 3M only after 15 days, further improvement during 6 months follow up did not significantly change MOD group: significant progressive improvement was observed after 15. day, 2 months and 6 months in 1M and 3M but not in DML SEV group: significant improvement was only observed in DML values Correlation between postoperative clinical findings and postoperative EDX was not assessed.
Glowacki et al.	1996	167 patients (227 hands) 93 patients underwent EMG, NCV; no EMG/NCV: 101 hands;	EMG, NCV	**positive group (99 hands):** 89/99 - positive Phalen's test, 50/99 - positive Tinel's sign, 88/99 - nocturnal numbness and paraestesias, 94/99 - symptoms with daily activities; **negative group (27 hands):** 26/27- positive Phalen's test, 16/27 - positive Tinel's sign, 26/27 - nocturnal numbness and paraestesias, 24/27 - symptoms with daily activities	**Complete relief:** 64/99 - positive EMG/NCV; 16/27 - negative EMG/NCV 61/101 - group without preoperative EDX; **Patrial relief:** 28/99 - positive EMG/NCV 9/27 - negative; EMG/NCV,	7/99 - positive EMG/NCV; 2/27 - negative EMG/NCV; 7/101 - no EMG/NCV;	InStat 2.0 software (Graphpad Software, San Diego, CA) - to determine if there is any difference in outcome between the three EMG/NCV groups	There was no significant differences between positive EMG/NCV and negative EMG/NCV group in postoperative results.

Concannon et al.	1997	349 patients, 460 hands, **group 1**: 62/460 **group 2 (2A+2B)**: 398/460 hands	EMG	**normal EDX** 62/460 **mild-moderate EDX** 348/460 **severe EDX** 50/460	relief of symptoms: group 1: 61/62 hands (98%); group 2A+2B: 390/398 (98%);	complication rate: group 1: 3/62 hands(5%); group 2A+2B: 20/398 (5%)	**Test for age differences with respect to the maximal EDX classification:** A one-way analysis of variance **Examining the association with gender:** Chi-square test	There was no significant difference among three groups in complication rate and degree of success of surgical release.
Padua et al.	1997	**group A** (normal DML) 9/53 hands; **group B** (DML = 4-6 ms) 32/53 hands; **group C** (DML > 6 ms) 12/53 hands	**1M** - median NCV thumb to wrist; **3M** - median NCV middle finger to wrist; **DML** - distal motor latency wrist to thenar eminence	**Symptoms:** numbness: A 22.2%, B 87.5%, C 100% nocturnal awakening: A 88.9%, B 90.6%, C 83.3%; **Objective deficits:** sensory deficits: A 11.1%, B 21.9%, C 66.7%; motor deficits: A 0%, B 18.8%, C 100%; thenar atrophy: A 0%, B 34.4%, C 100%	<u>15th day</u>: paraestesias and pain: A 77.8%, B 93.8%, C 58.3%; <u>6th month</u>: **Symptoms** numbness: A 100%, B 87.5%, C 66.7%; nocturnal awakening: A, B and C 100% **Objective data:** sensory deficits: A 100%, B 96.9%, C 66.7%; motor deficits: A 100%, B 96.9%, C 41.7%; thenar atrophy: A 100%, B 84.4%, C 58.3%	**15th day: paraestesias and pain:** A (2/9), B (2/32), C (5/12); **6th month**: **Symptoms** numbness: B 12.5%, C 33.3%; **Objective data:** sensory deficits: B 3.1%, C 33.3%; motor deficits: B 3.1%, C 58.3%;	**Determination of difference between pre- and postoperative data:** Wilcoxon matched pairs test	Group A: significant postoperative improvement was observed in 1M and 3M tests in 6 month follow-up, no significant change was in postoperative DML results Group B: postoperative significant improvement was observed in 1M, 3M, DML in 6 month follow-up Group C: significant improvement was observed in 1M, 3M, DML results 6 months after operation Correlation between postoperative clinical findings and postoperative EDX was not assessed.
Choi et al.	1998	mild (48/294); moderate (142/294); severe (104/294)	**DML** - 8-cm distal motor latency, **DSL** - 14-cm distal sensory latency,distal sensory amplitude	**duration of symptoms before diagnosis and CTR:** < 3 months 24/294 hands, < 1 year 73/294, < 5 years 94/294, < 10 years 60/294, > 10 years 43/294	**within 1 week**: mild 33/48 (68.8%), moderate 97/142 (68.3%), severe 68/104 (65.4%); **within 3 months**: mild 11/48 (22.9%), moderate 28/142 (19.7%), severe 20/104 (19.2%); **within 1 year**: mild 3/48 (6.3%), moderate 12/142 (8.5%), severe 14/104 (13.5%);	mild 1/48 (2.1%), moderate 5/142 (3.5%), severe 2/104 (1.9%);	**Chi-squared test:** recovery from symptoms after operation, Patient Satisfaction with the Operation	There was significant differences among 5 symptom-duration groups in relief of symptoms ($p<0.05$) There was no significant difference between mild, moderate and sever groups in rate of recovery from symptoms after operation

								(p>0.05), and Patient Satisfaction with the Operation (p>0.05)
Nakamura et al.	1999	**Mild** 2/40 hands **Moderate** 13/40 hands **Severe** 25/40 hands	DML, SCV	no data	40/40 hands achieved improvement after 12 months; **Recovery rate (%):** at the post-operative **1st month:** 18.0±9.0, **1st-3rd months:** 12.8±8.6, **3rd-6th months:** 6.0±6.0; **6th-12th months:** 8.1±10.0	5/40 hands – complications (dysesthesiae)	Paired t-test	Significant difference was observed between 1st -3rd months and 3rd-6th months during postoperative follow-up (p<0.05) Correlation with post-operative improvements in subjective symptoms was not assessed.
Dudley Porras et al.	2000	3/85 group 1 (<1 SD from normal values), 6/85 (>1 SD from normal values), 22/85 group (> 2 SD from normal values), 55/85 group 4 (> 3 SD from normal values)	DML, SNCV, EMG, SNAP	52/86 (60%) Tinel's sign positive, 64/86 (75%) Phalen's sign positive, 51/86 (59%) - Durkan's sign positive; thenar atrophy 15/86 (17%); **subjective test** (mean) 3.4 (range 1.1-5) points; **Functional scale** (mean) 3.2 (range 1.1-5) points	1/3 - group 1, 3/6 group 2, 14/22 group 3, 50/55 - group 4; **mean values±SD: subjective test** 1.4 (range 1-5) points (P<0.001), **Functional scale** 1.4 (range 1-4.2) points (P<0.001),	2/3 group 1, 3/6 group 2, 8/22 group 3, 5/55 group 4	**Pearson's correlation coefficient** - assessment of the relationship between types of quantitative variables; **Student's t-test**: comparison between pre- and postoperative means for the functional scales and subjective tests	There were significant differences between pre- and postoperative results in subjective test (P<0.001), functional test (P<0.001), SNCV (P<0.001) and there was no significant improvement between pre- and postoperative DML, motor amplitude and sensory amplitude values among all patients. Significant correlation was found only between symptom severity and functional status outcomes (r=0.58, P<0.001)
Mondelli et al.	2001	5 clinical stages; 2 severity scales: Padua's severity scale (1997), Mondelli's scale (1997)	**MCV, DML** (with CMAP), **SAPa** - sensory action potential amplitude of the median nerve	**Total BQ score** (mean value): 5.9±1.5 (range 2.5-9.4). **Mean electrophysiologic al sensory scale:** 3.5±1.2 (range 1-5); **Mean electrophysiologic al motor scale:** 2.5±1.1 (range 1-5); **Padua's**	**1st month follow-up:** Total BQ score 3.8±1.1 (range 2-7.9) **Electrophysiol sensory scale** 2.8±1.4 (range 0-5), **Electrophysiol motor scale** 2.1±1 (range 1-5), **Padua scale** 2.8±1 (range 1-5);	**1st month follow-up:** 22/104 – minor complications **6th month follow-up:** 3/104 hands has symptom severity score the same or worse than before the operation	**Spearman test** - correlations between age, the clinical severity score, the electrophysiologic al scales and the BQ scores	There was not a significant correlation between improvement of electrodiagnostic studies and postoperative BQ scores. There were a significant improvements between pre- and

				**electrophysiological scale:** 3.1±0.8 (range 1-5);	**6th month follow-up:** Total BQ score 3.1±1.1 (range 2-7.3) **Electrophysiol sensory scale** 2.2±1.1 (range 0-5), **Electrophysiol motor scale** 1.8±0.9 (range 1-5), **Padua scale** 2.4±1 (range 1-5)			postoperative EDX result (P<0.001) and between pre- and posoperative BQ scores (P<0.001).
Finsen et al.	2001	16/68 - normal EDX results, 52/68 - abnormal EDX,	no data	data of 63 patients (with well response after surgery), **Pain or paraesthesia in radial fingers** 63/63; **Phalen's test positive** 58/63; **Woken at night** 56/63; **Tinel's sign positive** 46/63	63/68 - **well response;** 52/68 - rapid resolution after surgery;	**5/68 patients;** 2 patients - categorized as false positive diagnosis of the CTS	Chi square and Wilcoxon's non-parametric tests	There was no significant difference between both groups. In some cases, the neurophysiological studies were normal with evident no improvement after surgery.
Bland et al.	2001	**grade** 0: 86/1240; 1: 65/1240; 2: 161/1240, 3: 335/1240; 4: 185/1240; 5: 292/1240; 6: 116/1240	palm/wrist median/ulnar comparison, ring finger "double peak", median SCV, median DML, CMAP from APB	no data	**success rate:** **grade 0** (86/1240): 51.2%, **grade 1** (65/1240): 64.6%, **grade 2** (161/1240): 75.8%, **grade 3** (335/1240): 77.0%, **grade 4** (185/1240): 74.1%, **grade 5** (292/1240): 66.4%, **grade 6** (116/1240): 46.6%;	**failure rate: grade 0:** (86/1240) 13.0%, **grade 1:** 15.0%, **grade 2:** 11.0%, **grade 3:** 8.0%, **grade 4:** 6.0%, **grade 5:** 13.0%, **grade 6:** 14.0%	**Kruskal–Wallis chi-squared test**– to determine difference between length of follow-up periods; **Logistic regression model** for prognosis in carpal tunnel decompression	According to logistic regression model, 5 factors were found to be significant for prediction of postsurgical outcome: age, preoperative NCS grade, gender, symptom score, duration of symptoms. There was nonlinear correlation between NCS grade and outcome.
Kouyoumdjian et al.	2002	**Group 0** 8/114 **Group 1** 76/114 **Group 2** 19/114 **Group 3** 9/114 **Group 4** 7/114	**median DSL, median DML, MR -** sensory median-radial difference	no data	**Complete relief "cure":** Group 0: 7/8, Group 1: 74/76, Group 2: 17/19, Group 3: 9/9, Group 4: 7/7	**Unchanged/ Worsed:** Group 0: 1/8, Group 1: 2/76, Group 2: 2/19, Group 3: 0/9, Group 4: 0/7	Fisher test frequency of "cure" vs unchanged/worsed	There was a significant difference between surgical and non-surgical treated group in frequency of complete relief (P<0.01). NO other significant differences were observed.
Borisch et al.	2003	**no grading, 237 patients (307 hands) without advanced neurophysiologic**	**DML** and **CMAP** recording over the APB	**Group A**: brachial nocturnal pain 111/159 (70%), paraesthesia 139/156 (89%),	**3rd month: Group A**: Relief of symptoms: brachial nocturnal pain 105/107	**1 patient of group B -** recurrence of nocturnal pain **3rd month: Group A:**	The linear regession analysis	There was no a significant difference between types of surgical procedure or

		**al changes (DML < 11 ms); group A -** simple open decompression (160 patients), **group B -** decompression with epineurotomy and visualization of the motor branch of the median nerve (147 patients)		Thenar atrophy 73/157 (47%), **Group B**: brachial nocturnal pain 95/146 (65%), paraesthesia 136/146 (93%), Thenar atrophy 57/148 (39%)	(98%), paraesthesia 99/108 (92%), Thenar atrophy 62/105 (59%), **Group B:** brachial nocturnal pain 85/93 (91%), paraesthesia 77/93 (83%), Thenar atrophy 57/92 (62%)	brachial nocturnal pain 2/107 (2%), paraesthesia 9/108 (8%), Thenar atrophy 43/105 (41%), **Group B:** brachial nocturnal pain 8/93 (9%), paraesthesia 16/93 (17%), Thenar atrophy 35/92 (38%),		preoperative NCS results in postoperative DML (P=0.35) and posoperative SCV (P=0.52)
Schrijver et al.	2005	176 patients, 156 completed questionaires 12 months after randomization, 138 underwent repeated NCS; no grading	**SNCV** (index finger), **DSL** (index finger), **median-ulnar DSL-difference** (ring finger), **DML**	**median values of questionnaires scores:** symptom severity score (1-5): 2,5 (interquartile range 1.8-3.0); functional status score (1-5): 2.1 (interquartile range 1.5-2.9)	**12th month (156 questionnaires):** symptom severity score (1-5): 1.1 (interquartile range 1-1.5, mean change 1.1±0.8); functional status score (1-5): 1 (interquartile range 1-1.4, mean change 0.9±0.9);	no data	**Spearman rank correlation coeffitients** - for relationships between values of NCSs and different outcome measurements for complaints 12 months after randomization	There was significant improvement in NCS results and clinical outcome after surgery (P<0.001). There was found correlation between "number of nights waking up due to symptoms" and median-ulnar DSL difference 12 months after randomization (Spearman rank correlation coefficient 0.33, P 0.001). There was weak correlation between changes in NCS parameters and changes in outcome measures for complaints (range 0.00 to 0.26).
Tay et al.	2006	27 patients with moderate (15) and severe CTS (12) underwent surgery	Motor and sensory median-ulnar responses comparison, median DML, median DSL	no data	**3rd month: complete resolution:** 1/15 patient of moderate group and 3/12 patients of severe group ; **improvement:** 7/15 patients of moderate group and 7/12 patients of severe group	**3rd month: unchanged:** 6/15 patients of moderate and 2/12 patients of severe group; **worse**: 1/15 patient of moderate and 0/12 patients of severe group	no data	No statistical analysis was done Any significant change cannot be confirmed
Malladi et al.	2009	191 patients, severity of the CTS was based on	**To obtain CSI 3 sensory NCSs were used;**	no data	**Outcomes after surgical release grouped by CSI:**	**Outcomes after surgical release grupped by**	chi-square analysis, with P < 0.05 considered a	Patients with median CMAP amplitude >9.0

		CSI: 6/191 CSI <1.0, 50/191 CSI 1.0-2.4, 49/191 CSI 2.5-4.6, 48/191 CSI > 4.6, 38/191 Absent CSI	**Median motor nerve conduction studies:** CMAP; DML		Complete resolution of symptoms: CSI normal (<1.0): 3/6, CSI 1.0-2.4: 25/50, CSI 2.5-4.6: 35/49, CSI> 4.6: 26/48; CSI absent: 14/38;	**CSI: Persistent pain and/or parasthesiae: CSI normal (<1.0):** 3/6, **CSI 1.0-2.4:** 25/50, **CSI 2.5-4.6:** 14/49, **CSI> 4.6:** 22/48; **CSI absent**: 24/38	sigfinificant difference.	mV tended to have the best outcome and there was a correlation between CMAP amplitude and relief of paraesthesia (P=0.05) but no correlation with absence of pain (P=0.096). There was a significant association between relief of paraesthesia and DML (P=0.006) but not for resolution of pain (P=0.24).
Inukai et al.	2012	**3 classes based on Padua's classification: extreme CTS** (CTS-A 8/65, CTS-B 9/65), **severe CTS** 14/65, **moderate CTS** 34/65	**2L-IN -** second lumbrical interossei test, DML, SCV, APB-CMAP, 2L-CMAP, INT-CMAP	Mean duration of symptoms: extreme CTS-A: 30.8±25.5 months, extreme CTS-B: 28.2±12.7 months, severe CTS: 26.1±8.55 months, moderate CTS: 4.3±1.8 months	Clinical results: **Excellent:** 0/8 extreme CTS-A, 0/9 extreme CTS-B, 3/14 severe CTS, 24/34 moderate CTS; **Good:** 0/8 extreme CTS-A, 6/9 extreme CTS-B, 8/14 severe CTS, 10/34 moderate CTS; **Fair :** 5/8 extreme CTS-A, 3/9 extreme CTS-B, 3/14 severe CTS, 0/34 moderate CTS	**Poor outcome (unchanged or worsened symptoms):** 3/8 extreme CTS-A, 0/9 extreme CTS-B, 0/14 severe CTS, 0/34 moderate CTS	Statistical Package for the Social Sciences software (version 15.0, SPSS, Chicago, IL, USA).	No statistical analysis between pre- and postoperative results was done. Any significant change cannot be confirmed.
Tahririan et al.	2012	**None (normal)** 0/17; **mild CTS** 0/17 hands **mild to moderate** 0/17 hands **moderate CTS** 4/17 hands **moderate to severe:** 2/17 hands; **severe CTS** 11/17 hands	DML, SCV, DSL	no data	**Satisfaction frequency: 6th month**: **5/17 completely satisfied** (postoperative EDX severity: 1/2 - Mild severity, 3/3 - mild to moderate severity, 1/7 - moderate severity) **12th month: 7/17 - completely satisfied** (postoperative achieved CTS severity: 7/8 - mild EDX)	**Satisfaction frequency: 6th month:** 5/17 - **completely dissatisfied** (3/3 with severe CTS) **12th month: 4/17 - completely dissatisfied** (1/3 - normal EDX, 1/3 - moderate CTS, 2/2 - severe CTS)	SPSS v.16 ; a one-tailed P value < 0.05 was considered statistically significant	The process of improvement in each parameters was statistically significant at both time points using repeated measure analysis of variance. (p<0.05).
Beck et al.	2013	78 patients, 72 underwent EDX testing; grading according	DML	75/78 – numbness 70/78 – pain 65/78 - night pain/numbness	**Clinical postoperative evaluation**:	**persistent night pain/numbness**	Fisher exact and Wilcoxon rank sum tests	There was no significant improvement in pain, nocturnal

		to DML: **mild** 28/72; **moderate** 18/72, **severe** 26/72		50/78, Phalen's positive 38/78, thenar atrophy	70/70 - **relief of pain;** 61/65 - **relief of night pain/numbness**: 9/21 mild NCS, 12/13 moderate NCS, 26/26 severe NCS 65/75 **- relief of numbness** 21/27 mild NCS, 15/16 moderate NCS, 25/26 severe NCS	**:** 2/21 - mild NCS, 1/13 - moderate NCS, 0/26 - severe NCS; **persistent numbness:** 6/27 mild NCS, 1/16 moderate NCS, 1/26 severe NCS		pain/ numbness, numbness between all three groups (P=0.31 and P=0.09, respectively).
Fowler et al.	2015	**grading according Beck et al. (2013): mild** 11/56; **moderate** 22/56, **severe** 23/56	DML	**Nocturnal symptoms were present** 50/56 (89%); **constant daytime numbness** 28/56 (50%);	**48 hours postoperatively**: **Daytime numbness and/or tingling:** 6/10 mild CTS, 16/22 moderate CTS, 9/23 severe CTS; **Nocturnal symptoms**: 9/11 with mild CTS, 12/18 with moderate CTS, 16/21 with severe CTS.	**first post-operative appointment** (10–14days after surgery) 2/11 (18%) mild CTS, 2/22 (9%) moderate CTS, 13/23 (57%) severe CTS	Multivariate analysis - to determine the most important predictors of resolution of symptoms	Severity of CTS based on NCS and two-point discrimination test were found to be significant predictors of postoperative outcome.
Kronlage et al.	2015	47/95 **- moderate CTS,** 48/95 - **severe CTS,** according to the AAEM modified criteria for diagnosis of CTS	EMG, NCV	**Moderate CTS group:** CTSS score: 2.7 (2.4-2.9), CTSS numbness: 2.7 (2.4-3.0) , CTSS pain: 2.6 (2.2-2.9); **Severe CTS group:** CTSS score: 3.1 (2.9-3.4), CTSS numbness: 3.2 (3.0-3.5), CTSS pain: 3.1 (2.8-3.3)	46/47 **moderate group,** 39/48 **severe group**. Symptoms improved in both groups from the preoperative assessment to the 2-week postoperative assessment (P < .001),	1 patient of moderate group, 9 patients with severe CTS	multivarable linear reggression	There was found weak to moderate associations between higher preoperative symptom severity—higher preoperative CTSS scores and symptom severity after surgery. Severe CTS was the only factor significantly correlated with total CTSS score and CTSS numbness 3 months after surgery. Age and CTS severity were significant prognostic factors of CTSS pain score.
De Kleermaeker et al.	2017	39 patients, normal EDX studies	DIG-4, DIG-1; PALM-test -, DML from APB	atrophy of the APB: 1/39 (2.6%), **weakness:** APB 4/37 (10.8%), opponens pollicis muscle 2/37 (5.4%), **Disturbed**	23/33 (70.0%) important improvement, 13/33 (39.4%) completely asymptomatic; mean±SD:	2/33 (6.1%) - deterioration	SPSS Statistics 24.0. Diferences in SSS and FSS after follow-up were performed with a paired t test in case of nominal	There was a significant improvement in SSS and FSS outcome in patients treated surgically in

				**sensibility:** two-point discrimination 31/37 (83.8%), monofilament 17/37 (45.9%), Tinel positive 24/38 (63.2%), Phalen's positive 28/38 (73.7%); 27 patients (mean ± SD): SSS 2.95 ± 0.64, FSS 2.19±0.72	SSS 1.86±0.95, FSS 1.80±0.89; Difference: SSS -1.09±1.11 (P<0.001), FSS -0.39±1.02 (P=0.055)		distribution, and the Wilcoxon signed rank test for non-nominal distribution; Chi-square test - comparison in baseline characteristicts	comparison with conservatively treated patients (p=0.036).
Rivlin et al.	2018	criteria established by Werner and Andary: **mild** 20/256 hands (8%), **moderate** 126/256 hands (49%), **severe** 110/256 hands(43%)	EMG	**QuickDASH:** Mild: 45 (21-64), Moderate 40 (25-60), Severe 42 (23-58); **SSS**: Mild: 2.4 (1.6-2.7), Moderate: 2.5 (1.8-2.9), Severe 2.4 (1.7-3.1) **FSS** Mild: 1.6 (0.63-2.7), Moderate: 1.9 (1.0-2.8), Severe: 2.0 (1.7-3.1).	**3rd month: QuickDASH**: Mild 18 (15-28), Moderate: 7.5 (2.5-25), Severe 13 (5.0-28); **SSS**: Mild: 0.82 (0-1.3), Moderate: 0.36 (0.07-0.91), Severe: 0.73 (0.27-1.4); **FSS:** Mild: 0.63 (0-1.5), Moderate 0.38 (0-1.0), Severe: 0.50 (0.13-1.4). Postoperative pain improvement occurred regardless of EDX grade	no data	bivariate analysis	There was found positive correlation between moderate EDX grade, female sex, younger ange (<60 years old) and greater improvement.
